# A Facile and Scalable Approach in the Fabrication of Tailored 3D Graphene Foam via Freeze Drying

**DOI:** 10.3390/ma14040864

**Published:** 2021-02-11

**Authors:** Tony Thomas, Arvind Agarwal

**Affiliations:** Plasma Forming Laboratory, Mechanical and Materials Engineering, Florida International University; Miami, FL 33174, USA; tonthoma@fiu.edu

**Keywords:** freeze drying, reticulated foam, graphene nanoplatelet, process thermodynamics

## Abstract

One of the challenges in the processing of advanced composite materials with 2D reinforcement is their extensive agglomeration in the matrix. 3D architecture of 2D graphene sheets into a Graphene Foam (GrF) assembly has emerged as an effective way to overcome agglomeration. The highly reticulated network of branches and nodes of GrF offers a seamless pathway for photon and electron conduction in the matrix along with improved mechanical properties. 3D GrF nano-filler is often fabricated by chemical vapor deposition (CVD) technique, which demands high energy, slow deposition rate, and restricting production to small scale. This work highlights freeze-drying (FD) technique to produce 3D graphene nanoplatelets (GNP) foam with a similar hierarchical structure to the CVD GrF. The FD technique using water as the main chemical in 3D GNP foam production is an added advantage. The flexibility of the FD in producing GNP foams of various pore size and morphology is elucidated. The simplicity with which one can engineer thermodynamic conditions to tailor the pore shape and morphology is presented here by altering the GNP solid loading and mold geometry. The FD 3D GNP foam is mechanically superior to CVD GrF as it exhibited 1280 times higher elastic modulus. However, thermal diffusivity of the FD GNP foam is almost 0.5 times the thermal diffusivity of the CVD GrF due to the defects in GNP particles and pore architecture. The versatility in GNP foam scalability and compatibility to form foam of other 1D and 2D material systems (e.g., carbon nanotubes, boron nitride nanotubes, and boron nitride nanoplatelets) brings a unique dimensionality to FD as an advanced engineering foam development process.

## 1. Introduction

**Figure 1:**
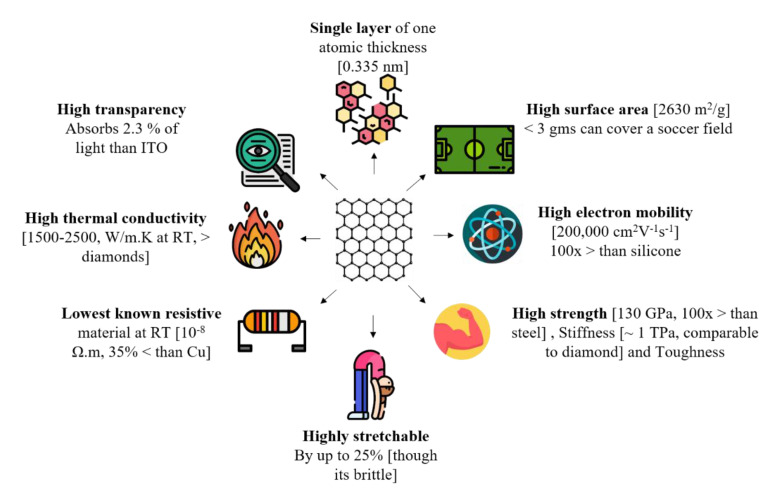
Schematic detailing the properties of pristine graphene.

Over the past five decades, extensive progress has been achieved in understanding the effect of reinforcing materials in enhancing the matrix properties [1,2,3,4]. Reinforced composite materials are broadly adapted in all engineering applications due to a unique combination of material properties they can manifest, for example lightweight yet strong, thermally insulating yet electrically conducting, and tailored fracture mechanics. Reinforcement materials are categorized into fiber, flakes, particulates, and fillers [5]. The choice of reinforcement materials is based on the desired intrinsic property enhancement in the material matrix. For example, particulates are not effective in enhancing the toughness or fracture resistance in a material, as they randomly orient themselves in the material matrix. Typically, flakes or two-dimensional (2D) platelet material forms of reinforcement improve the composites’ physical and mechanical properties. 2D platelets are packed parallel to one another, resulting in higher density packing than fiber reinforcements. Hence, 2D reinforcements can produce composite materials with boosted material properties. Carbonaceous nanomaterials have shown promising potential as a reinforcement material in developing advanced engineering composites. Graphene (Gr), a 2D material, which is a single layer of sp^2^ hybridized carbon atom, has inspired material researchers and industry owing to its impressive arsenal of properties, as summarized in Figure 1 [6,7,8].

**Figure 2:**
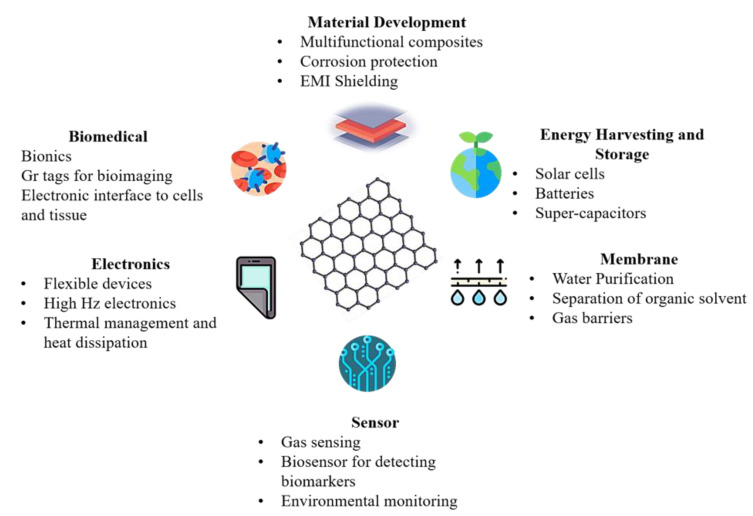
Schematic summarizing applications of graphene (Gr)-based materials.

Gr offers ~3 times the specific surface area than the carbon nanotubes (CNT), and hence its addition has proved to improve the overall material properties such as strength, stiffness, and thermal and electrical conductivity [9]. Such augmentation in the matrix material properties due to Gr addition has led to its potential application in energy devices, semiconductors, heat sinks, protective coating, etc. Figure 2 summarizes potential applications of Gr based materials [6,7,10].

**Figure 3:**
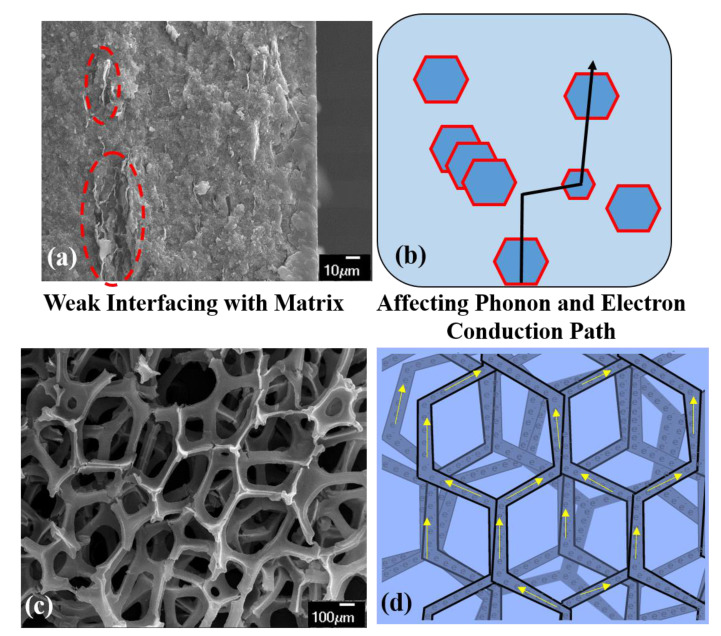
(**a**) SEM image showing clustered and poorly dispersed 2D Gr flakes (highlighted in red dashed oval) in a ceramic matrix; (**b**) schematic representing the discontinuous contact between poorly dispersed 2D Gr in the material matrix, impeding phonon and electron conduction; (**c**) SEM image of pristine GrF; and (**d**) schematic representing how the architecture of GrF offers a seamless pathway for phonon and electron conduction.

2D Gr sheets have a higher tendency to agglomerate due to intermolecular π–π bonding [11]. These agglomerates form clusters that can restack to form graphitic structure and act as a stress concentrator leading to an adverse effect on the mechanical properties. Moreover, lack of homogeneous distribution of 2D Gr in the non-conducting (both thermal and electrical) material matrix, as shown in Figure 3a [low temperature co-fired dense ceramic reinforced with graphene nano-platelets (GNP)] produced by spark plasma sintering), causes a discontinuity in the contact between Gr flakes, as represented in Figure 3b, affecting the phonon and electron conduction path, thus limiting the thermal and electrical conductivity in the composite [12,13]. Although various physical and chemical dispersion techniques such as sonication, surface functionalization, and ball milling have been employed to overcome the agglomeration, Gr tends to align randomly. Other strategies such as crumbling the graphene sheets and adding spacers have also been explored to overcome the agglomeration challenge [14,15].

**Figure 4:**
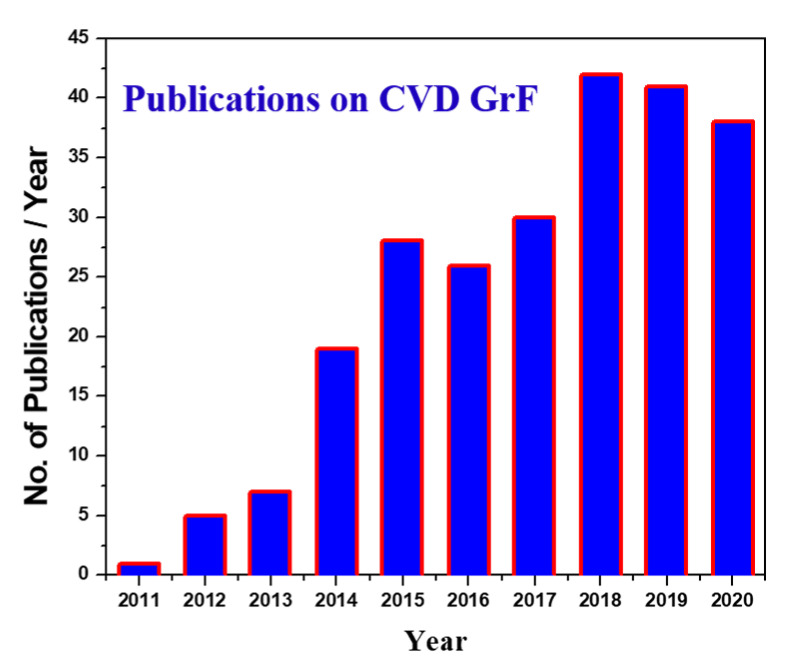
Data showing the number of scientific publications on GrF produced by chemical vapor disposition (CVD) since its advent in 2011.

Graphene foam with a highly reticulated hierarchical structure, as shown in Figure 3c, has proven to be an effective solution against agglomeration and restacking of 2D Gr flakes. This macroporous, free-standing, 3D graphene foam (GrF) has emerged as a prevalent nanofiller material [16,17,18,19,20]. 3D GrF has a homogenous microstructure and can be readily introduced into a low viscous material matrix without complicated dispersion techniques. The reticulated architecture comprises an interconnected network of branches and nodes, as shown in Figure 3d, which facilitates an uninterrupted pathway for phonon and electron conduction, unlike the 2D Gr flakes (refer to Figure 3b). The pristine free-standing GrF shown in Figure 3c offers filler properties such as ultra-low density (<4 mg/cm^3^), enhanced surface area (~850 m^2^/g), and improved electron and phonon conduction due to reduced inter Gr sheet contact resistance [21,22,23,24]. As a result, 3D GrF has been used in producing several composite materials for applications such as scaffolds [21], strain sensors [16], vibration dampeners [12,25], supercapacitors [26], fracture-resistant materials [27], and thermal interfacing [28]. The pristine 3D GrF is often fabricated via a template-directed chemical vapor disposition (CVD) technique [24,25,26,27,28]. Since its advent in 2011, from the trend analysis of scientific publications on CVD GrF (Figure 4, data from the web of science), it is evident that there is a tremendous interest in tapping into intrinsic properties of graphene arranged in a 3D hierarchical structure. 

**Figure 5:**
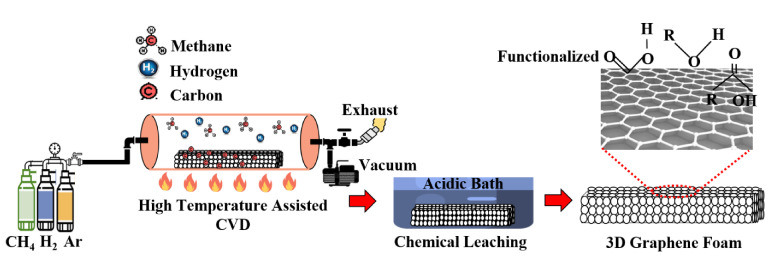
Schematic showing the production of 3D GrF by CVD technique [29,30,31].

The procedure of producing 3D GrF by CVD technique is shown in the schematic in Figure 5 [29,30,31]. A porous metal foam with a reticulated structure, typically nickel with the desired pore shape and size, is chosen as the graphene deposition template. A hydrocarbon gas, usually methane (CH_4_), is used as the carbon source. At a high temperature of 1000 °C, carbon released from the methane decomposition is deposited on the porous metal template. Due to a catalytic reaction, thin layers of Gr films are precipitated on the nickel template. Once the required layers of Gr film are deposited on the template, a poly methyl methacrylate (PMMA) is deposited on the precipitated Gr film. PMMA prevents the Gr network’s collapse upon removing the metal template by chemical etching, as shown in Figure 5. Hydrochloric or ferric chloride acid is the typical etchant used. Finally, the etched GrF foam structure undergoes an acetone bath to dissolve the PMMA and obtain a monolithic 3D GrF with a highly reticulated system [24].

Although the CVD technique produces high-quality GrF with controlled layer numbers and crystallinity [24], it has some limitations. One of the main challenges in producing 3D GrF with CVD is scalability, as the size of the foam is limited to the reaction chamber capacity. Having the metal substrate with the desired architecture brings further challenges. The additional challenge includes obtaining a completely uniform graphene layer on the substrate, as the kinetics of gas diffusion change within the reaction chamber’s volume [32,33]. This affects the chemical reactions on the metal substrate. The current CVD technique to produce free-standing GrF requires specialized equipment and is a highly energy driven process as it demands elevated temperature for chemical reactions [24]. The byproducts of the process, such as reaction gases, are extremely combustible. The metal etchant acid used to separate the substrate from Gr film is corrosive and toxic. Special reformers are required to neutralize these toxic byproducts. As a result, the pristine GrF produced is expensive, and not easily scalable. To address the limitations mentioned above and the disadvantages of the CVD technique in the fabrication of GrF, an eco-friendly process known as Freeze Drying (FD) is explored in this research. The procedure involves freezing an aqueous suspension, followed by sublimation of the frozen aqueous solvent at low pressure to obtain porous architecture of suspended materials. A variety of materials can be subjected to FD, which suggests that the process relies on the physical interaction of the slurry materials rather than their chemical interactions, unlike CVD [34,35,36,37].

Over the past two decades, FD has emerged as a promising technique to fabricate porous materials with high surface area for biomedical, structural, and energy applications [34,35,36,37,38]. FD was first developed as a single-step forming process to produce dense ceramic and only later it was realized that the thermodynamic control of the freezing vehicle could yield porous material with hierarchical structure [39]. In FD, the particles in the slurry are rejected from the propagating crystal front, collecting the particles between the growing crystal front. Thus, by controlling the heat transfer rate and direction, isotropic or anisotropic solidification can be introduced in the slurry system to get directional pores with uniform size or random pores of varying size [40]. The solidification time influences the pore size and the wall thickness. Solidification time increases linearly with the thickness of the sample [34]. Hence, optimal temperature control and control of formulation is paramount to obtain a 3D foam structure with isotropic properties. Many graphene and graphene based composite 3D foam structures of various chemical binders and dispersants have been produced by FD for applications such as flexible supercapacitors, electromagnetic shielding, sensors and catalyst support [40,41,42,43,44,45].

Hence, herein the FD technique’s simplicity is demonstrated in artificially stacking the graphene nanoplatelets (GNP) into a 3D hierarchical structure similar to the CVD produced GrF. The FD process’s simplicity in designing 3D GNP foam with controlled pore size and morphology is explored as this is the main challenge with the CVD technique. Here, two methodologies were used successfully to produce GNP foam with different pore size and morphology: (i) regulating the heat transfer rate by changing the amount of GNP (solid loading) in the aqueous slurry; and (ii) controlling the heat transfer direction by changing the mold geometry. The 3D GNP foam produced by FD is compared with the CVD GrF to understand the effect of processing on the mechanical and thermal properties. This study also demonstrates that 3D foams of other nanomaterials can be produced by the simple, eco-friendly, and cost-effective FD technique.

## 2. Experimental

### 2.1. Slurry and 3D GNP Foam Preparation

Graphene nanoplatelets used in this study were purchased from XG Science, Lancing, MI, USA (Avg. particle size 15 µm and thickness of 6–14 nm, bulk density 0.0–0.1 g cm^−3^). Deionized water was the primary solvent used. Carboxymethyl cellulose (CMC, ~1.1 wt.%, Mw ~700,000, Millipore Sigma, Burlington, MA, USA) was used as the dispersant. Styrene-butadiene rubber (SBR, ~2.1 wt.%, MTI Corporation, Richmond, CA, USA) was the binder used to hold the GNP particles together once the solvent is sublimed. Two slurry compositions were prepared to regulate the pore size by controlling the length of the solidifying waterfront. Two mold shapes, circular and rectangular, were used to regulate the pore morphology by controlling the heat transfer direction and rate. Both the molds were made from Aluminum (Al). Initially, CMC and SBR were vortex mixed in DI water for 30 min to obtain the solvent. GNP slurry batches of 8 wt.% and 9.5 wt.% was vortex mixed in the solvent (~89 wt.% and 87 wt.% respectively de-ionized water) for 120 min. The slurry was frozen in the Al molds for 4 h at −56 °C and dried (sublimation of water) for 24 h in 1 Pa ambient pressure in a freeze drier (Pro-Freeze Dryer PLT300, Pro-Lab Inc, Fort Worth, TX, USA).

### 2.2. Microstructure Analysis

A field emission scanning electron microscope (FE-SEM JEOL JSM-6330F, JEOL Ltd. Tokyo, Japan) was used to analyze the FD GNP foam’s pore size and morphology. An acceleration voltage of 20 kV was used throughout the secondary electron imaging. The pore size was evaluated from the SEM images using ImageJ 1.52a an open source image processing tool.

### 2.3. Compression Test

Displacement controlled compression tests were performed on FD GNP foam and commercially available CVD GrF (Graphene Supermarket, Calverton, NY, USA). All samples were strained to 55% of their original length at a displacement rate of 0.016 mm/second using Electroforce 3200 mechanical tester (Bose corporation, Eden Prairie, MN, USA) equipped with a 25 N load cell. Both FD (8 wt.% GNP, cast in Al mold) and commercial CVD GrF had dimension of 10 mm in diameter and a thickness of ~1.2–1.5 mm. They were sliced into required dimensions using a razor blade.

### 2.4. Thermal Analysis

Thermal diffusivity (α) of the FD GNP foam and commercial CVD GrF (Graphene Supermarket, Calverton, NY, USA, USA) across the thickness (in-plane) was measured by laser flash technique (NETZSH LFA 467 HT HyperFlash, Selb, Germany) at temperatures between 25 and 75 °C. Both FD (8 wt.% GNP, cast in Al mold) and commercial CVD GrF had dimension of 10 mm in diameter and a thickness of ~1.2–1.5 mm.

### 2.5. Raman Analysis

Micro-Raman spectroscopy analysis was performed using Spectra-Physics 3900S (Newport Corporation, Irvine, CA, USA) equipped with Ti-sapphire crystal (514 nm) as the target. The Spectra-Physics also consists of a detector with 4 cm^−1^ spectral resolution from Kaiser Optical Systems, Inc. (Ann Arbor, MI, USA), a laser power (18 mW), with a spot size of 2 µm.

## 3. Results and Discussion 

### 3.1. Controlling the Foam’s Pore Size and Morphology

#### 3.1.1. Solid Loading

**Figure 6:**
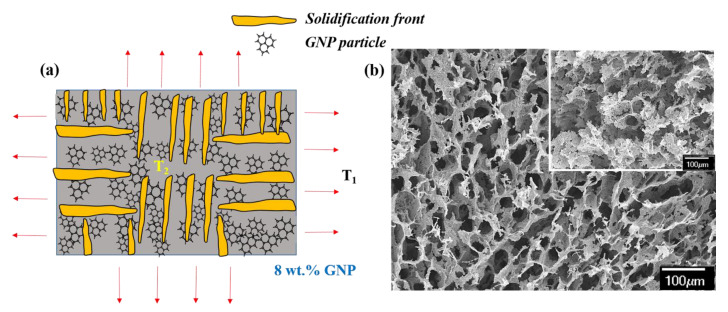

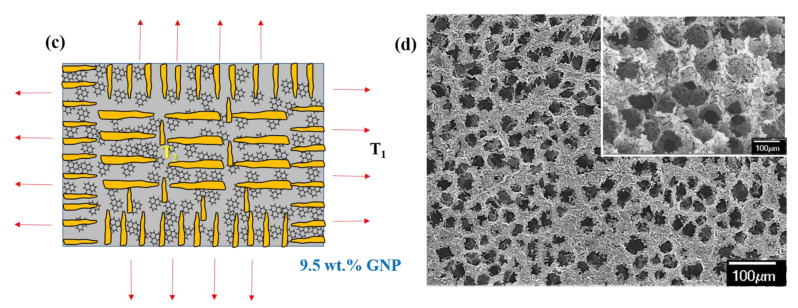
(**a**,**c**) Schematic representing the influence of free energy of the slurry system on the length and diameter of the freezing ice crystal; (**b**) SEM image of 3D GNP foam with 8 wt.% GNP; and (**d**) SEM image of 3D GNP foam with 9.5 wt.% GNP (3D foam made in a circular Al mold of 20 mm length, 10 mm diameter, and 0.16 mm thick).

To comprehend the FD technique’s theoretical understanding, one should consider the system’s thermodynamic free energy as a critical parameter [38]. Since the process involves solidifying the colloidal slurry (GNP mixed in DI water, CMC, and SBR solvent), the freezing solvent front must segregate the GNP particles as they propagate. For the GNP particles to be pushed away from the propagating ice crystal, the free energy of the system ∆σ must be positive. If ∆*σ* is negative, the GNP particles will be trapped inside the freezing ice crystal, inducing the formed foamy structures’ low structural integrity. Hence, a slurry for FD should satisfy the following thermodynamic condition, which is given by Equation (1) [36]:

(1) $$\Delta \sigma =\sigma_{sp}-(\sigma_{lp}+\sigma_{sl})>0$$

where *σ_sp_*, *σ_lp_*, and *σ_sl_* are the interfacial free energies related with the solid_(ice-crystal)_–particle _(GNP)_, liquid _(DI water)_ –particle _(GNP)_, and solid_(ice-crystal)_–liquid _(DI-water)_ interfaces, respectively. If Equation (1) is satisfied, then the solidification front rejects the GNP particles in the slurry system. If the system’s overall free energy decreases (become negative), then, based on Equation (1), the particles in the slurry are engulfed by the propagating ice crystal, which is not ideal for developing porous 3D foam with a hierarchical structure. The solid loading is one such variable that drastically affects the ∆σ of the system. ∆*σ* is directly influenced by the thermal gradient generated in the slurry system, which dictates the freezing ice crystals’ growth rate or kinetics. The temperature gradient induced in a slurry system can be represented as Equation (2) [36]:

(2) $$\frac{\left(T_{1}-T_{2}\right)}{L}$$

where *T_1_* and *T_2_* are the time-dependent temperatures of the atmosphere outside the mold and inside the mold (GNP slurry), respectively, as shown in the schematic in Figure 6a,c. *L* is the length of the mold along which the majority of the heat transfer takes place. The practical demonstration of Equations (1) and (2) is shown in Figure 6. Figure 6b is the 3D GNP foam produced with 8 wt.% GNP and Figure 6d is the 3D GNP foam made with 9.5 wt.% GNP. Solid loading was selected based on a general guideline that slurry with solid loading <5 vol.% and >45 vol.% often results in 3D porous structures with random pore size and morphologies [38]. As mentioned above, the solid loading affects the ∆*σ* of the slurry system, which alters the propagating ice crystals’ kinetics, influencing the size of the pores formed.

3D GNP foam with 8 wt.% GNP (Figure 6b) has an average pore size of ~110 µm with oblong pore morphology, while 3D GNP foam with 9.5 wt.% GNP (Figure 6d) has an average pore size of ~50 µm with near-circular pore morphology. In the slurry with 8 wt.% GNP, the magnitude of ∆*σ* is higher than slurry with 9.5 wt.% GNP. With the increased GNP content in the slurry, the thermal conductivity of the slurry increases. As a result, T_1_ equilibrates with T_2_ at a higher rate (low ∆*σ*), forming ice crystals with a smaller radius and length (refer to Figure 6c) that manifests into pores of smaller size and thick wall, as shown in Figure 6d and its respective inset. Increased pore walls or strut thickness is the result of mass conservation [46,47]. In the slurry with 8 wt.% GNP, its thermal conductivity is comparatively lower than 9.5 wt.% slurries. In this case, the slurry system experiences a thermal gradient for a prolonged period (high ∆*σ*), resulting in a longer ice crystal with a higher radius (refer to Figure 6a). After sublimation, the resultant foam will have increased pore size with a thin wall, as shown in Figure 6b and its respective inset. Researchers have also looked into other complex methods to better control the pore size and morphology by regulating the viscous stresses and pressure drops experienced by the particles as it interacts with the propagating solidification front [48].

#### 3.1.2. Effect of Mold Geometry on Pores

Another easily adaptable technique in tailoring desired pore size and morphology is by using molds of different geometry. The mold into which the slurry is poured is the critical source to impact the heat transfer between the freezing atmosphere and the slurry during solidification. This heat transfer is mainly convective type and takes the form, Equation (3):

(3) $$Q=h_{c}A(T_{2}-T_{1})$$

where *Q* is the heat transferred per unit time, *A* is the mold surface area for the heat transfer, *h_c_* is the heat transfer thin film co-efficient of the heat transfer medium, *T*_2_ is the initial mold surface temperature, and *T*_1_ is the temperature of the freezing environment.

**Figure 7:**
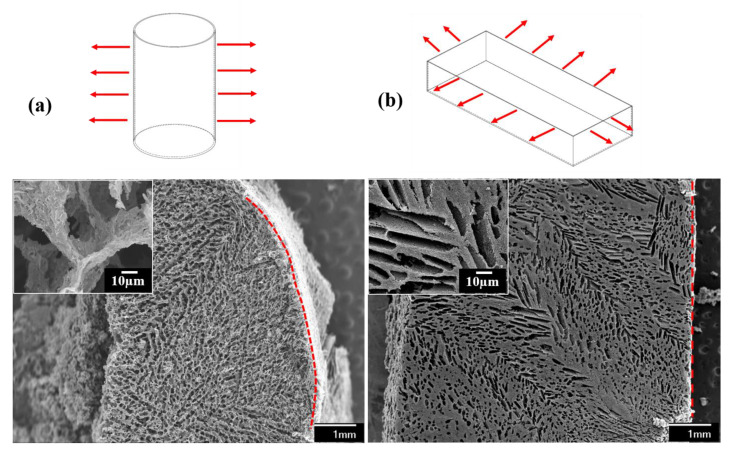
(**a**) 3D GNP foam produced in a cylindrical mold (20 mm in length, 10 mm in diameter and mold thickness of 0.16 mm); and (**b**) 3D GNP foam made in a rectangular mold (50 mm in size, 20 mm in width, 10 mm in height diameter, and mold thickness of 0.16 mm). Both insets show the wall thickness (8 wt.% GNP slurry used in both the molds).

The solidification conditions control pore morphology and size as described in the previous section. The ice crystals grow parallel but in the opposite direction to the thermal gradient (red arrows in Figure 7 represent the heat transfer direction). In the cylindrical mold (Figure 7a), most of the heat transfers along the radial surface (radial surface area ~640 mm^2^).

Hence, the ice crystals grow from the circumference of the mold inwards. Therefore, in the SEM in Figure 7a, pores can be observed aligned along the rim (represented by the red dashed curve in Figure 7a and radially grown inwards. In addition, there is heat transfer from the bottom of the mold (surface area ~80 mm^2^). However, heat transfer along the length of the mold is not as dominant as across the circumference. Here, the thermal gradient can be considered as homogenous, resulting in oriented hierarchical porous architecture. Due to higher heat transfer along the mold’s circumference, pore channels run through the entire sample forming a reticulated porous structure. In the rectangular mold (drawing in Figure 7b), there is a heterogeneous thermal gradient as the entire mold surface area is of significant dimension. Along the length, the surface area is ~500 mm^2^_,_ the surface area is ~200 mm^2^ along the width, and the bottom mold surface area is ~1020 mm^2^. As seen in the SEM in Figure 7b, the difference in a thermal gradient across the rectangular mold surface resulted in ice crystals’ growth in a random direction. Although long-range lamellar porous structures can be observed, they do not form a reticulated system with a continuous pore channel. Localized small range lamellar structures can also be observed in the SEM in Figure 7b. Unlike in cylindrical mold, the pores are not aligned along the edges of the rectangular mold (red dashed line in Figure 7b), but, instead, they are at an angle due to the difference in a thermal gradient along the length, width, and height of the mold. Therefore, the temperature field must be carefully controlled to obtain homogenous pore size and morphology in FD samples. In CVD, the pore size and morphology depend on the metal template used to deposit the graphene film. Hence, using CVD processing, tailoring the pore morphology and size in the 3D reticulated GrF is not straightforward as in the FD technique. The new porous metal substrate with the desired pore shape and morphology needs to be fabricated every time, whenever customization is required, which is later etched away, leading to wastage of metal and generation of toxic acid byproducts. 

**Figure 8:**
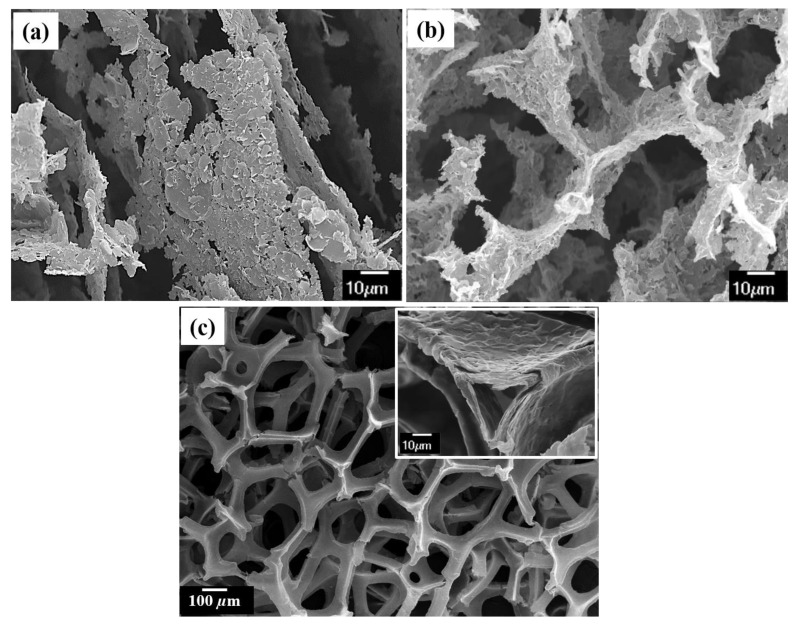
SEM images showing: (**a**) FD 3D GNP foam with basal plane aligned; (**b**) reticulated 3D architecture of FD GNP foam with solid wall or strut; and (**c**) reticulated 3D architecture of CVD GrF with hollow wall or strut (inset showing hollow wall or strut).

Another advantage of producing 3D GNP foam by FD is obtaining a foam with aligned GNP along its basal plane. Since the direction of the solidification crystal front’s growth can be controlled, the GNP’s basal plane aligns along the solidification direction, as shown in Figure 8a. With such a reinforcement nanofoam, it is possible to produce advanced engineering materials with directional properties, such as a material system that conducts electrons along the thickness, preferably across the material’s bulk. In the case of CVD produced GrF, although one can obtain high purity GrF with a preferred number of graphene films, as the basal plane rests on the deposited surface, they are at different angles concerning each other. Hence, producing 3D GrF with the aligned basal plane is challenging via the CVD technique. Figure 8c shows a pristine 3D GrF produced by the CVD technique, and the respective inset shows the wall or the strut of CVD GrF. It can be seen that the CVD technique results in foam with a hollow strut due to the removal of the metal substrate by acid etching, whereas 3D GNP foam produced via FD technique has foam with solid strut with highly oriented GNP, as shown in Figure 8b, of similar strut architecture to CVD GrF. FD GNP foam packs higher GNP content in a given strut as compared to the CVD produced GrF.

### 3.2. Mechanical and Thermal Properties of Graphene Foams

Since GrF is an emerging nanofiller used to augment the mechanical and phonon conduction properties of the matrix material, the FD GNP foam’s mechanical and thermal properties are analyzed and compared with CVD GrF. The FD GNP used in the property characterization has 8 wt.% of GNP and was cast in a cylindrical mold, as shown in Figure 7a.

**Figure 9:**
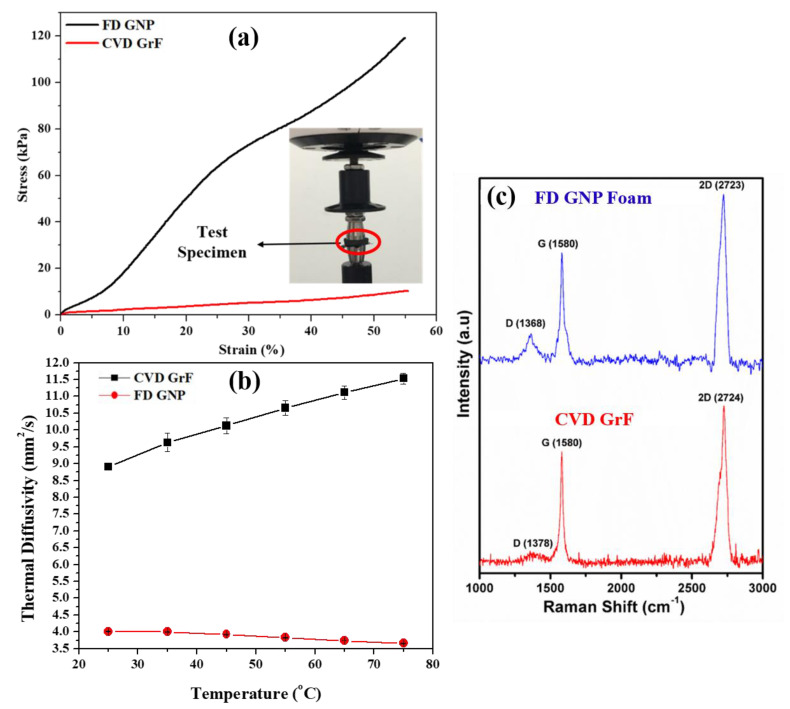
(**a**) Compressive stress vs. strain plot comparison of FD GNP foam and CVD GrF; (**b**) thermal diffusivity of FD GNP foam vs. CVD GrF; and (**c**) Raman spectra of FD GNP foam and CVD GrF.

**Figure 10:**
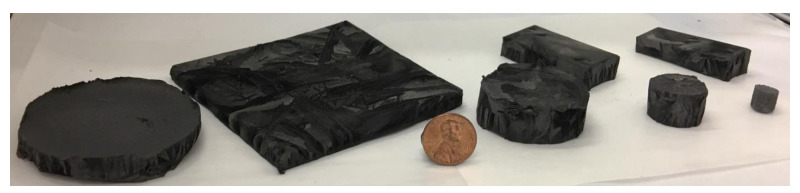
3D GNP foam produced in various sizes, shapes, and thicknesses by FD technique to highlight the scalability of the process with ease.

Figure 9a is the compressive stress vs. strain plot of FD GNP foam and CVD GrF. The inset in Figure 9a shows the test sample (within the red oval) placed between the flat plates of the mechanical testing machine. The sample merely has a large surface area compared to the surface area of the flat plate used in the compression test. Approximately 0.15 mm^2^ of the sample area is not in contact with the flat plate. FD GNP foam records a compressive strength of ~120 kPa with an elastic modulus of 283 ± 33 kPa, whereas CVD exhibited a compressive strength of ~10 kPa and an elastic modulus of 0.22 ± 0.024. FD GNP foam exhibits ~12 times the compressive strength and ~1280 times more elastic modulus than CVD GrF. It should be noted that the GNP foams in this study were not subjected to any type of heat treatment process after FD to increase its rigidity. The increase in the mechanical properties can be attributed to the presence of a solid wall architecture (refer Figure 8b) exhibited by FD GNP foam unlike CVD GrF, where the wall is hollow (refer Figure 8c). Trace amount of polymeric rubber in FD GNP foam also contributes to the increase in mechanical properties. The substantial increase in the slope of the FD GNP foam with increased stress can be due to the efficient stress transfer along the node–branch architecture of the foam and bending of the solid struts of the foam [25,28]. Other reasons for strengthening in graphene-based foams can be the flattening of intrinsic corrugations, inter layer van der Waals spring-like actions, kink band formation, and membrane vibrations [25,28]. It is well known that pristine CVD GrF is challenging to handle, as even a minuscule force, as small as the soft touch of a human, disintegrates the foam. As a result, integrating CVD GrF into a highly viscous material matrix without compromising its hierarchical structure is challenging. The use of large CVD GrF (the foam buckles due to its weight and disintegrates) is still a challenge. Thus, commercially available pristine CVD GrF are small with a typical size of 2 inches × 2 inches and a thickness of 1.2 mm. The presence of a small amount of elastomeric binder in FD GNP foam produces a rigid foam that can be easily cut into any shape and thickness. The production of FD GNP foam of various shapes, sizes, and thicknesses with controlled pore morphology and directionality, as shown in Figure 10, is possible with ease, and no special tooling or equipment is needed.

Figure 9b is the thermal diffusivity exhibited by FD GNP foam and CVD GrF in the temperature range 25–75 °C. CVD GrF has 2.25 times higher thermal diffusivity than FD GNP foam at 25 °C, and it increases to three times at 75 °C. The elastomeric binder that increased FD GNP foam’s mechanical property is the one of the reasons for the decreased thermal diffusivity. SBR has a negative glass transition temperature (−60 °C), and, above this temperature, the thermal conductivity of SBR decreases asymptotically [49]. Hence, the thermal diffusivity of the FD GNP foam decreases with increased temperature. Another reason for decreased thermal diffusivity is due to the lack of solid-phase continuity across the strut wall of the FD sample (refer to Figure 8a,b). Raman spectroscopy was employed to evaluate the graphene defects in both foams, as defects can influence the intrinsic phonon conduction property [24]. Table 1 tabulates the intensity of D, G, and 2D peaks deduced from the Raman spectra shown in Figure 9c.

The ratio of I_D_/I_G_ shines a light on the extent of defect in graphene. Table 1 shows that GNP used in FD GNP foam has three times more defects than graphene forming the CVD GrF. This resulted in the distortion of the phonon conduction band in FD GNP foam, as confirmed by the I_2D_/I_G_ ratio, which is 1.1 times higher than that of CVD GrF. The higher number of defects and distortion of the phonon conduction band in the GNP used in FD GNP foam can also explain the reduced thermal diffusivity in FD GNP foam.

Due to its chemical purity, CVD GrF is advantageous for high thermal conductivity and small-scale non-structural applications. However, when the nano-filler 3D graphene foam’s structural strength is essential, FD GNP foam is a great alternative. Although using FD GNP foam, the thermal conductivity may be compromised to a certain extent due to the polymeric binder, the benefits, such as particle alignment and tailorable pore size, morphology, and scalability, make FD GNP foam versatile in this class of nano-fillers. This work provides an outlook on the FD process in producing novel reinforcement material with a 3D hierarchical structure. One of FD’s primary advantages over the CVD technique in producing 3D foam structures is that the FD process is independent of material chemistry, unlike CVD. Hence, any material with proper slurry formulation can be artificially stacked into hierarchical reticulated structures (Figure 10).

**Figure 11:**
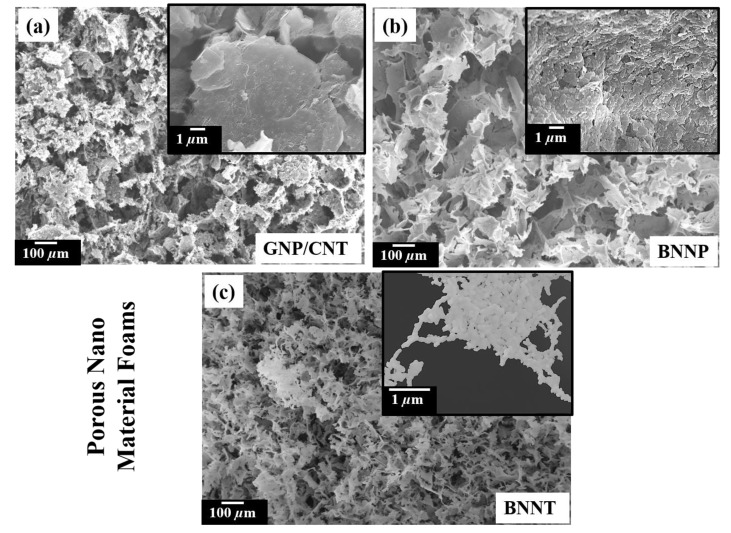
Porous 3D Foam produced by FD: (**a**) GNP/CNT composite foam; (**b**) BNNP foam; and (**c**) BNNT foam. Insets show the wall features of the foams.

Figure 11 demonstrates that any material system, whether 1D, 2D, or 3D, can be cast into highly reticulated structures easily without the need for any expensive equipment. Figure 11a shows GNP/CNT foam’s SEM image with the GNP decorated by CNT (shown in the inset). Figure 11b is the SEM image of 2D hexagonal boron nitride platelet (BNNP) foam with a thin leaf-like wall made up of highly aligned BNNP particles (refer to inset in Figure 11b). Finally, Figure 11c shows 1D hexagonal boron nitride nanotubes (BNNT) formed into porous structures of untangled BNNT (refer to inset in Figure 11c). All the 3D structures shown in Figure 11 were produced using similar slurry formation chemicals described in Section 2.1 (more details can be obtained upon request). Using the energy-efficient FD technique, the material science community can produce various novel engineering materials that can open multiple engineering applications such as 3D scaffolds for tissue engineering, high-density energy storage devices, high strength lightweight composite structural materials, and many more.

## 4. Conclusion

FD’s versatility in producing mechanically rigid yet highly reticulated GNP foam is presented. The FD process is advantageous when controlling the pore size and morphology of GrF. Using FD, without any need for specialized equipment, the intrinsic thermodynamics in the freezing solvent’s solidification can be engineered to achieve control over the reticulated architectural GNP foam design. In this work, the critical thermodynamic parameter that dictates the freezing ice crystal’s length and radius, free energy, was regulated by formulating slurry with different GNP solid loading and varying geometry molds. It was demonstrated that both these approaches influence the extent of thermal gradient and heat transfer direction to produce GNP foam with controlled pore size and morphology. A unique feature in the GNP foam made by FD is the alignment of GNP’s basal plane along the direction of the propagating ice crystal. This can induce intrinsic directional properties of GNP in the reinforced material matrix. In addition, unlike CVD produced GrF, the FD produced GNP foam walls are solid and not hollow, providing high graphene density for similar wall thickness. Compression test of FD GNP foam exhibited ~1280 times higher elastic modulus than CVD GrF, reassuring that FD GNP foam is a rigid and robust reinforcing nano-filler that would not disintegrate or collapse when infiltrated with high viscous slurry for new material development. Although FD GNP foam’s thermal property is 0.5 times the CVD GrF, the ease of process scalability and ability to artificially stack any material into a highly reticulated 3D structure makes FD a unique foam producing technology.

## Figures and Tables

**Table 1:** The intensity of D, G, and 2D peak for FD GNP and CVD GrF foams.

Sample	I_D_	I_G_	I_2D_	I_D_/I_G_	I_2D_/I_G_
CVD GrF	873.55	3408	5378	0.081	1.453
FD GNP	364.37	4516	6565	0.26	1.57

## Data Availability

Data reported in this study can be obtained by requesting the authors.
